# Supplementary material for: Protocol for evaluation of a virtual wheelchair simulator in assessing mobility skills and cognitive abilities in diverse populations: A multicentric mixed-methods pilot study
Source: PLoS One. 2025 Jun 6;20(6):e0325186. doi: 10.1371/journal.pone.0325186 (PMC12143504; doi:10.1371/journal.pone.0325186)
Supplement: S1 Appendix — (DOCX) [file pone.0325186.s001.docx]

Title of the Study: Evaluation of a Virtual Wheelchair Simulator in Assessing Mobility Skills and Cognitive Abilities in Diverse Populations: A Multicentric Mixed-Methods Pilot Study

Principal Investigator: Debora Pereira Salgado

Contact Information: [d.psalgado@research.ait.ie](mailto:d.psalgado@research.ait.ie)/ [a00257244@tus.ie](mailto:a00257244@tus.ie)

The proposed study has the following supporting documents:

|  | Document Title | Description |
| --- | --- | --- |
| 1 | Study Protocol Checklist | An investigator checklist to ensure that all procedural steps are followed for the study. |
| 2 | Informed Consent Form | This form outlines the study's purpose, procedures, potential risks and benefits, confidentiality measures, and the voluntary nature of participation. It ensures that participants understand the study and provide informed consent before enrolling. |
| 3 | Participant Information Sheet | This document provides a plain language explanation of the study, including its objectives, procedures, potential risks, benefits, and contact information for the researchers. It helps participants understand the study before deciding to participate. |

**PROTOCOL CHECKLIST FOR THE TESTS:**

**Subject ID: _____ _____________________________ Gender: _____**

**Date: __/ / Start Time : _____ End time: _____**

| Phase | Steps | Status |
| --- | --- | --- |
| 1. Before the Test | | |
|  | Print induction documents (Information Sheet, Consent Form, and questionnaires) |  |
|  | Check data synchronization framework and verify synchronization |  |
|  | Check Wheelchair Simulator Software |  |
|  | Check Joystick Control |  |
|  | Check EEG (Headband) Data Collection Module |  |
|  | Check OpenFace & Webcam ( head and eye-landmarks Data Collection Module) |  |
|  | Measure battery levels of wristbands and headband |  |
| 1. During the Test | | |
|  | Pre-Briefing of the study |  |
|  | Obtain informed consent |  |
|  | Collect demographics information |  |
|  | Sensor placement (wristbands and headband) |  |
|  | Baseline physiologic metrics acquired |  |
|  | WST test |  |
|  | MoCA test |  |
|  | Previous experience with wheelchair simulator? |  |
|  | Simulator session (Session ID) |  |
|  | PAAS Assessment during the test |  |
|  | Monitor participant comfort |  |
|  | Post-experience questionnaire and interview briefing and completion |  |
| 1. After the Test | | |
|  | Return sensors |  |
|  | Backup data (wristbands, headband, and OpenFace records) |  |
|  | Charge batteries of sensors |  |
|  | Scan induction documents and save originals |  |
|  | Review recorded data for quality and anomalies |  |

**INFORMED CONSENT FORM**

Title of the Study: Evaluation of a Virtual Wheelchair Simulator in Assessing Mobility Skills and Cognitive Abilities in Diverse Populations: A Multicentric Mixed-Methods Pilot Study

Principal Investigator: Debora Pereira Salgado

Contact Information: [d.psalgado@research.ait.ie](mailto:d.psalgado@research.ait.ie)/ [a00257244@tus.ie](mailto:a00257244@tus.ie)

Introduction:

You are invited to participate in a research study designed to evaluate the effectiveness of a wheelchair skill assessment and training simulator in improving the mobility skills of wheelchair users. Before deciding whether to participate, it is crucial that you understand the study's purpose, procedures, potential risks, and benefits. Please read the following information carefully. If you have any questions or need further clarification, do not hesitate to ask before making your decision.

Study Purpose:

The purpose of this study is to investigate the impact of a virtual wheelchair training simulator and skill assessment application on the power mobility skills and confidence levels of wheelchair users. By participating, you will help advance knowledge in wheelchair training methods and skill assessment and may benefit from improved mobility skills and increased confidence in navigating various environments.

Procedures:

If you agree to participate, you will be asked to:

1. Demographic Information: You will be asked to provide basic demographic information.
2. Pre-Test Assessment: Your initial mobility skills will be assessed using standardized tests, such as the Wheelchair Skills Test (WST) and Montreal Cognitive Assessment (MoCA), to understand your current abilities before the training.
3. Simulator Training Program: You will participate in a structured training program using the wheelchair training simulator, where you will navigate virtual scenarios that replicate real-world environments and challenges.
4. Guidance and Support: During the simulator sessions, experienced instructors will provide guidance and instructions as you engage with each scenario.
5. Post-Test Feedback: After the training, you will take part in a feedback session, which includes both a questionnaire and open-ended interview questions. This feedback will help us gather your insights and experiences with the simulator program. We would also like your permission to record this interview to ensure accurate analysis.

Data Collection and Use of Wearables:

During simulator sessions, you may be asked to wear sensors that collect physiological data, and cameras may record movements of your head and eyes to analyse interactions with the virtual environment. All data will be anonymized and used exclusively for research purposes.

Risks and Benefits:

While there are minimal physical risks associated with participating in this study, you may experience mild discomfort or fatigue during the training sessions. However, these risks will be minimized by the presence of trained instructors who will guide you throughout the training process. The potential benefits of participating include improved mobility skills, increased confidence in wheelchair navigation, and contribution to the development of effective training methods for wheelchair users.

Confidentiality:

Your identity and personal information will be kept strictly confidential. Any data collected during this study will be anonymized and stored securely. Only authorized researchers involved in this study will have access to the data. Any data related to your identity and personal information will be discarded at the completion of this research.

Voluntary Participation and Withdrawal:

Participation in this study is entirely voluntary. You have the right to refuse participation or withdraw from the study at any time, without penalty or impact on your medical care. Your decision to participate or withdraw will not affect your current or future relationship with the research team or the institution.

Contact Information:

If you have any questions or concerns regarding this study, you can contact the principal investigator, Debora Pereira Salgado, at [d.psalgado@research.ait.ie](mailto:d.psalgado@research.ait.ie) or IWA’s manager Paul Ryan at [paul.ryan@iwa.ie](mailto:paul.ryan@iwa.ie)

| **Please Answer (YES OR NO) for the following statements:** | |
| --- | --- |
| 1. I am satisfied that I understand the information provided and have had enough time to consider the information. |  |
| 1. I do not suffer from photosensitive epilepsy or any other form of epilepsy. |  |
| 1. I’m not pregnant and/or I am not experiencing any symptoms of pregnancy. |  |
| 1. I have not consumed alcohol beverages for the last 24 hours. |  |
| 1. I slept at least 6 hours on the last 24 hours. |  |
| 1. I do not have physical limitations that prevent me from safely operating the wheelchair with a joystick. |  |
| 1. I do not have cognitive limitations that prevent me from understanding and answering the questionnaire attached to this form and following instructions while using the simulator. |  |
| 1. I do not have pre-existing medical conditions that could be exacerbated or worsened by the use of wheelchair simulator (e.g., uncontrolled seizures, or recent orthopaedic surgeries). |  |
| 1. I do not have a pre-condition for motion sickness or simulator sickness. |  |
| 1. I understand that my participation is voluntary and that I am free to withdraw at any time, without giving any reason, without my legal rights being affected. |  |
| 1. I understand that any data collected during this study will be used for research purpose only and in the strictest confidence. |  |
| 1. I confirm that I have more than 18 years old. |  |
| 1. I have consent from IWA’s clinical team to participate the above study. |  |
| 1. I agree to take part in the above study. |  |
| 1. I confirm that I have read the information sheet dated ___/___/__ for the above study and have had the opportunity to ask questions. |  |
|  | |

By signing this form, you indicate that you have read and understood the information provided, and voluntarily agree to participate in the study.

Participant's Name: _____________________________

Participant's Signature: _____________________________

Date: _____________________________

Researcher's Name: _____________________________

Researcher's Signature: ______________________________

Date: _____________________

**PARTICIPATION INFORMATION SHEET**

Title of the Study: Evaluation of a Virtual Wheelchair Simulator in Assessing Mobility Skills and Cognitive Abilities in Diverse Populations: A Multicentric Mixed-Methods Pilot Study

Principal Investigador: Debora Pereira Salgado

Contact Information: [d.psalgado@research.ait.ie](mailto:d.psalgado@research.ait.ie)/ [a00257244@tus.ie](mailto:a00257244@tus.ie)

Thank you for your interest in participating in our study. Before you make a decision, we would like to provide you with some important information about the study's purpose, procedures, and what your involvement would entail. Please take the time to read this information carefully. If you have any questions or concerns, feel free to contact us for clarification before making your decision.

Study Overview:

The aim of this study is to evaluate the effectiveness of a wheelchair training simulation in enhancing the mobility skills of wheelchair users like yourself. By participating, you will engage in a wheelchair mobility scenarios program that utilizes a virtual simulator to replicate real-life scenarios and challenges faced by wheelchair users.

Participant Involvement:

If you choose to participate, here is what your involvement in the study will entail:

1. Initial Assessment:

- You will undergo an initial assessment of your current mobility skills and cognitive abilities, including standardized tests such as the Wheelchair Skills Test and Montreal Cognitive Assessment (MoCA), administered by trained professionals.

2. Simulator Sessions:

- You will use a virtual reality wheelchair simulator, guided by experienced instructors who will support you throughout. You will perform tasks in various simulated scenarios that mirror real-life environments, allowing you to practice and potentially improve your mobility skills in a safe and controlled setting.

- Your participation may involve up to three sessions, depending on the study requirements.

3. Data Collection:

- During the study, data will be collected to evaluate the effectiveness of the training simulation. This may include objective measurements of your mobility skills, self-reported assessments of your confidence levels, and feedback regarding your experiences with the simulator.

4. Use of Wearables and Camera

- During the simulator session, you may be asked to wear devices that capture physiological data (e.g., wearable sensors) and cameras may be used to record head and eye movements to analyse your interactions with the virtual environment. All recordings will be used strictly for research analysis and will be anonymized.

5. Post-Experience Interview

- After the simulator session, we will conduct a post-experience interview to gather further feedback. With your permission, we would like to record this interview to ensure accurate annotation and analysis.

6. Confidentiality and Ethical Considerations:

- Your participation in this study will be treated with the utmost confidentiality. All data collected during the study will be anonymized and stored securely.

- Participation is entirely voluntary, and you have the right to withdraw from the study at any time without penalty or impact on your medical care or support services.

7. Potential Benefits and Risks:

- Potential Benefits: By participating in this study, you may experience improved mobility skills and increased confidence in navigating various environments. Your involvement will also contribute to the advancement of knowledge and the development of more effective training methods for wheelchair users.

- Risks: While there are minimal physical risks associated with the simulator training, you may experience mild discomfort or fatigue during the sessions. However, the presence of trained instructors will ensure that any risks are minimized, and your well-being is prioritized.

8. Contact Information:

- If you have any questions or concerns about the study or your participation, please feel free to contact us. We are available to provide additional information and address any queries you may have.

Thank you for considering participating in our study. Your contribution is invaluable, and we greatly appreciate your involvement in advancing wheelchair training methods.

Sincerely,

Debora Pereira Salgado

[d.psalgado@research.ait.ie](mailto:d.psalgado@research.ait.ie) / [a00257244@student.tus.ie](mailto:a00257244@student.tus.ie)
