# Supplementary material for: Protocol for evaluation of a virtual wheelchair simulator in assessing mobility skills and cognitive abilities in diverse populations: A multicentric mixed-methods pilot study
Source: PLoS One. 2025 Jun 6;20(6):e0325186. doi: 10.1371/journal.pone.0325186 (PMC12143504; doi:10.1371/journal.pone.0325186)
Supplement: S2 Appendix — (DOCX) [file pone.0325186.s002.docx]

Contents

[1. Pre-Experience Section 2](#_Toc181782541)

[1.1. Demographics Information 2](#_Toc181782542)

[1.2. Wheelchair Skills Test Questionnaire (WST-Q) Version for Powered Wheelchairs. 2](#_Toc181782543)

[1.3. Montreal Cognitive Assessment (MOCA) Version 8.1 English 2](#_Toc181782544)

[**2.** During Experience Section 3](#_Toc181782545)

[2.1. PAAS Scale 3](#_Toc181782546)

[2.2. Power Mobility Road Test Assessment Sheet 4](#_Toc181782547)

[**3.** Post-Experience Section 5](#_Toc181782548)

[3.1. NASA-TLX (Overall Cognitive Workload) 5](#_Toc181782549)

[3.2. Self-Assessment Manikin – SAM (Emotion) 7](#_Toc181782550)

[3.3. Usability, Immersion and Engagement 8](#_Toc181782551)

[3.4. Semi-structure Interview Section (exploring usability and potential applications of the wheelchair simulator system) 11](#_Toc181782552)

[4. Assessments Overview 13](#_Toc181782553)

# Pre-Experience Section

This section of the questionnaire gathers information about your background and baseline skills relevant to the wheelchair simulator study. Your responses will help us understand your experience with wheelchairs and any pre-existing cognitive or physical limitations that might influence your performance in the simulator.

## Demographics Information

| Questions | Information |
| --- | --- |
| Gender |  |
| Age |  |
| Duration of wheelchair use (years) |  |
| Wheelchair Type (e.g. Scooter or Power Wheelchair) |  |
| Joystick type (standard or any adaptation) |  |
| Dominant Hand |  |
| Diagnosis |  |
| Additional Information (optional) |  |

## Wheelchair Skills Test Questionnaire (WST-Q) Version for Powered Wheelchairs.

This questionnaire can be downloaded from <https://wheelchairskillsprogram.ca/en/skills-manual-forms/>.

## Montreal Cognitive Assessment (MOCA) Version 8.1 English

This questionnaire and training can be download from <https://mocacognition.com/>

# During Experience Section

To gain a deeper understanding of your experience using the wheelchair simulator, we'll be asking you to provide real-time feedback on your mental effort and perceived difficulty after completing each task. This information will help us evaluate the simulator's cognitive demands and its ability to replicate real-world challenges.

## PAAS Scale

**Mental Effort**


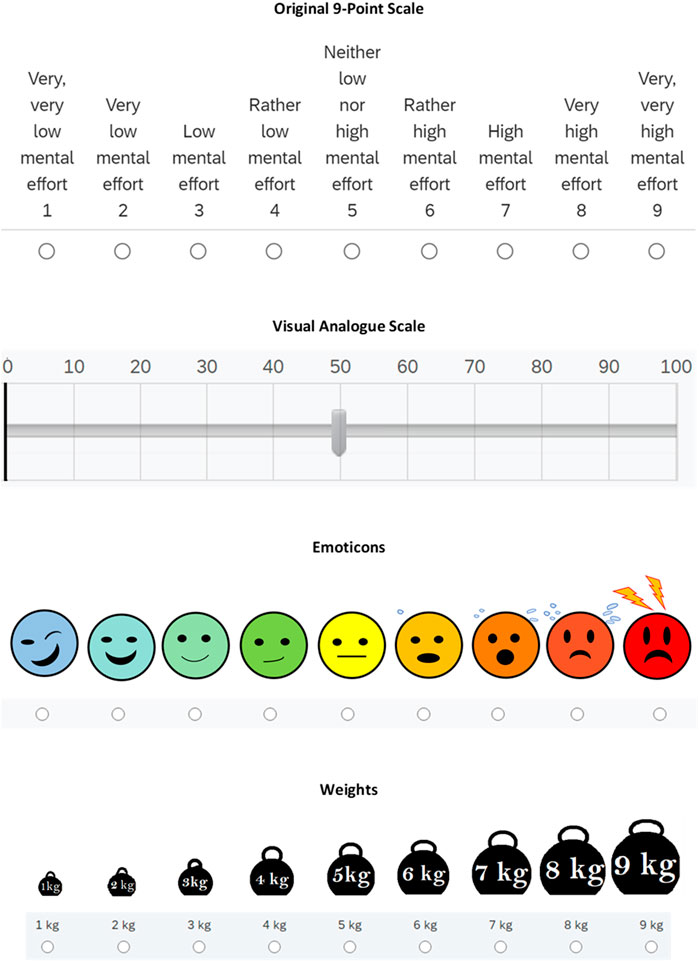


**Perceived Difficulty**


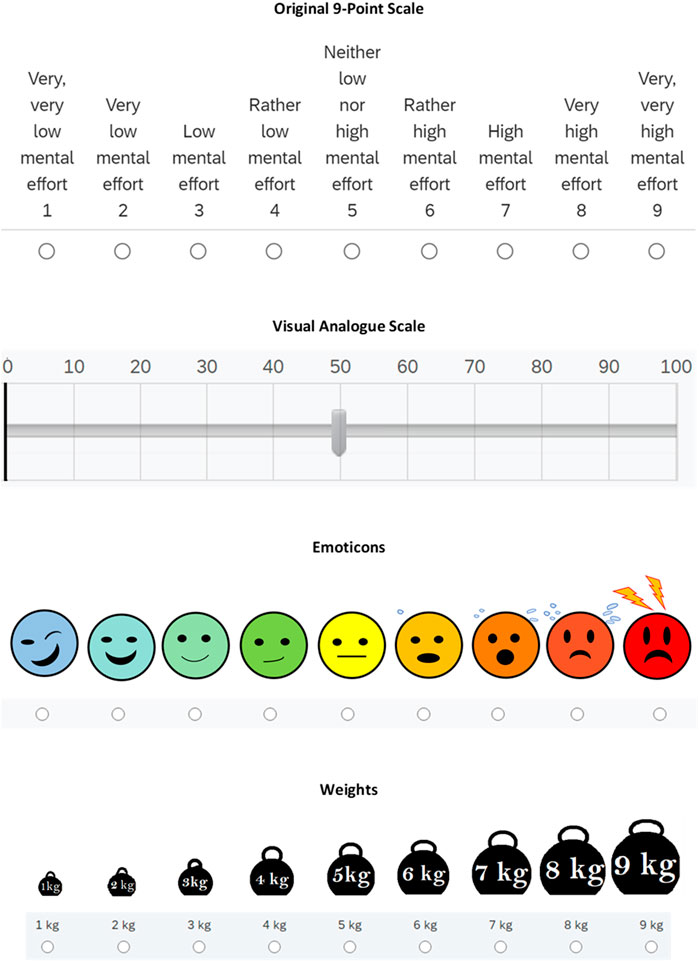


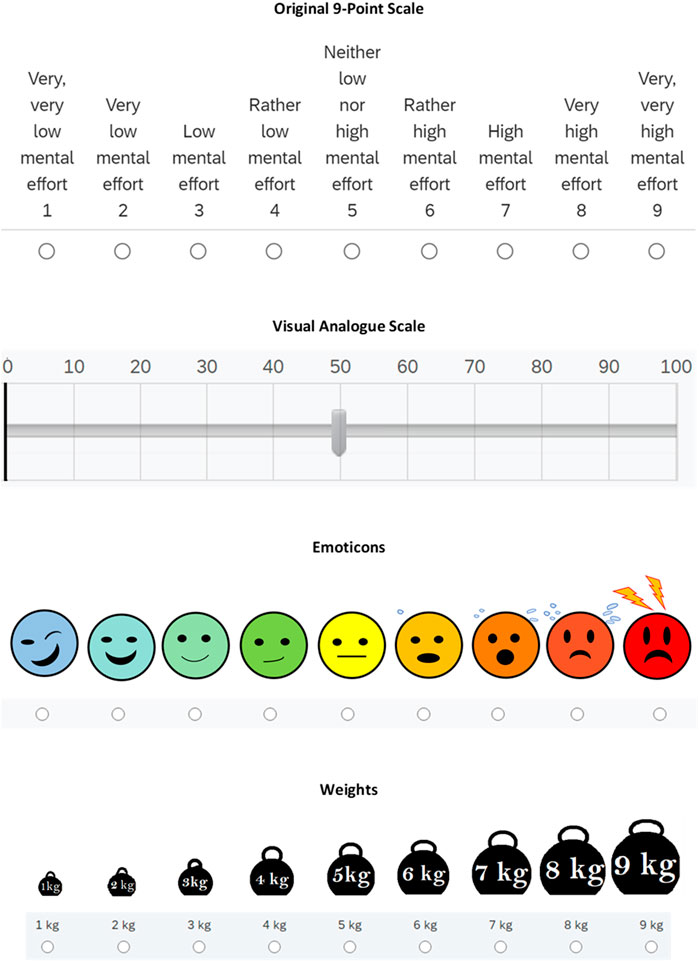


| Task | PAAS Scale | |
| --- | --- | --- |
|  | Mental Effort | Perceived Difficulty |
| 1 |  |  |
| 2 |  |  |
| 3 |  |  |
| 4 |  |  |
| 5 |  |  |
| 6 |  |  |
| 7 |  |  |
| 8 |  |  |
| 10 |  |  |
| 11 |  |  |
| 12 |  |  |

## Power Mobility Road Test Assessment Sheet

| **Element/Tasks** | **Score** | | | | **Comments** |
| --- | --- | --- | --- | --- | --- |
|  | **1** | **2** | **3** | **4** |  |
| 1. Approaching furniture |  |  |  |  |  |
| 1. Start and stopping wheelchair at will |  |  |  |  |  |
| 1. Crossing doorways without hitting walls |  |  |  |  |  |
| 1. Turning around a 90 right hand corner |  |  |  |  |  |
| 1. Turning around a 90 left hand corner |  |  |  |  |  |
| 1. Driving straight forward |  |  |  |  |  |
| 1. Driving straight backwards |  |  |  |  |  |
| 1. Turning 180 degrees |  |  |  |  |  |
| 1. Starting and stopping wheelchair |  |  |  |  |  |
| 1. Turning right and left upon command |  |  |  |  |  |
| 1. Driving straight forward in a narrow corridor not hitting walls |  |  |  |  |  |
| 1. Manoeuvre between objects |  |  |  |  |  |

**Score Definition:**

4 – Completely Independent: optimal performance, able to perform task in one attempt smoothly and safely.

3 – Completes task hesitantly, requires several tries, requires speed restriction, and/or bumps wall, objects, etc., lightly (without causing harm).

2 – Bumps objects and people in a way that causes harm or could cause harm to driver, other persons or to objects.

1 – Unable to complete task: reason:____________________________________. For example, may require verbal and/or visual cues or physical assistance.

# Post-Experience Section

Thank you for participating in the wheelchair simulator study. Your insights are invaluable in helping us understand how effectively the simulator reflects real-world wheelchair skills and provides useful assessments and training. To gain a better understanding of your experience with the wheelchair simulator, we invite you to answer a few brief questions. Your feedback will assist us in enhancing the simulator's user experience and overall effectiveness.

## NASA-TLX (Overall Cognitive Workload)

The evaluation you are about to perform is a technique that has been developed by Nasa to assess the relative importance of six factors in determining how much workload you experienced while preforming a task that you recently completed. These six factors are defined below on this page.

Read through them to make sure you understand what each factor means. If you have any questions, please ask your administrator.

| **Workload factors** | **Definition** |
| --- | --- |
| **Mental Demand Level (low/high)** | **How much mental add perceptual activity was required** (for example, thinking, deciding, calculating, remembering, looking, searching, etc)? Was the task easy or demanding, simple or complex, forgiving or exacting? |
| **Physical Demand Level (low/high)** | **How much physical activity was required** (for example, pushing, pulling, turning, controlling, activating, etc.)? Was the task easy or demanding, slow or brisk, slack or strenuous, restful or laborious? |
| **Temporal Demand Level (low/high)** | **How much time pressure did you feel** due to the rate or pace at which the tasks or task elements occurred? Was the pace slow and leisurely or rapid and frantic? |
| **Performance Level(good/poor)** | **How successful do you think you were in accomplish the goals of the task set by the experimenter** (or yourself)? How satisfied were you with your performance in accomplish these goals? |
| **Effort Level (low/high)** | **How hard did you have to work** (mentally and physically) to accomplish your level of performance? |
| **Frustration Level (low/high)** | **How insecure, discouraged, irritated, stressed**, and annoyed versus secure, gratified, content, relaxed, and complacent did you feel during the task? |

You will now be presented with a Series of rating scales. For each of the six scales, evaluate the task you recently performed by cross on the scale’s location that matches your experience. Each line has two endpoint that describe the scale. Consider your responses carefully in distinguishing among the different task conditions and consider each individually.

Mental Demand (How mentally demanding was the task?

How much mental and perceptual activity did you spend for this task?)


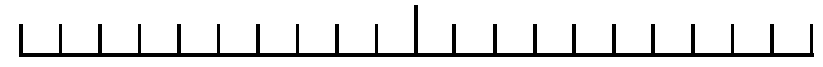


Very Low Very High

Physical Demand (How physically demanding was the task? How much physical activity did you spend for this task?)


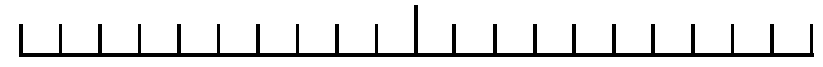


Very Low Very High

Temporal Demand (How hurried or rushed was the pace of the task?

How much time pressure did you feel in order to complete this task?


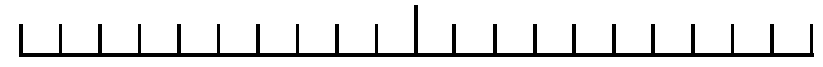


Very Low Very High

Performance (How successful were you in accomplishing what you were asked to do?

How successful do you think you were in accomplishing the goals of the task?)


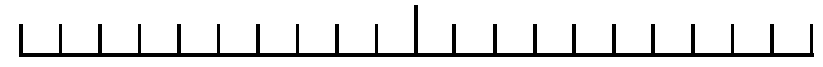


       Good   Poor

Effort (How hard did you have to work to accomplish your level of performance?)


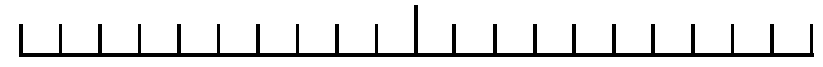


Very Low Very High

Frustration (How insecure, discouraged, irritated, stressed, and annoyed were you during this task?)


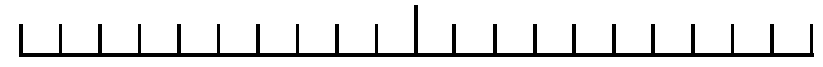


Very Low Very High

## Self-Assessment Manikin – SAM (Emotion)

You will be asked to rate your emotions towards to experience in using the Simulator.  It will be asked to rate on three separate scales.

**Rating Scales**

1. **Valence (Pleasant level)**

Pleasant Satisfied Neutral Unsatisfied Unpleasant


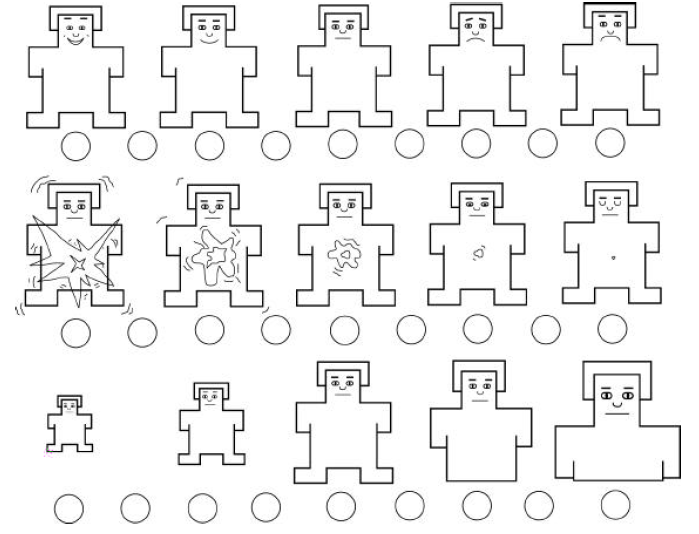


**2. Arousal (Excitement level)**

Excited Wide-awake Neutral Dull Calm


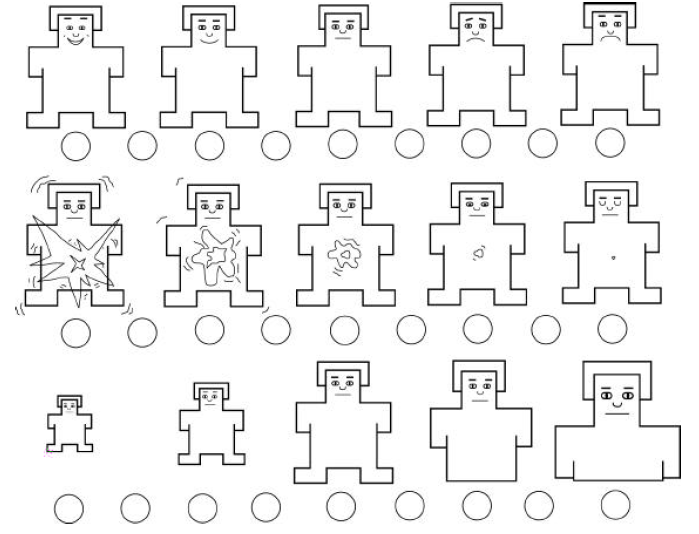


**3. Dominance (Emotion Control level)**

Dependent. Powerlessness Neutral Powerful Independent


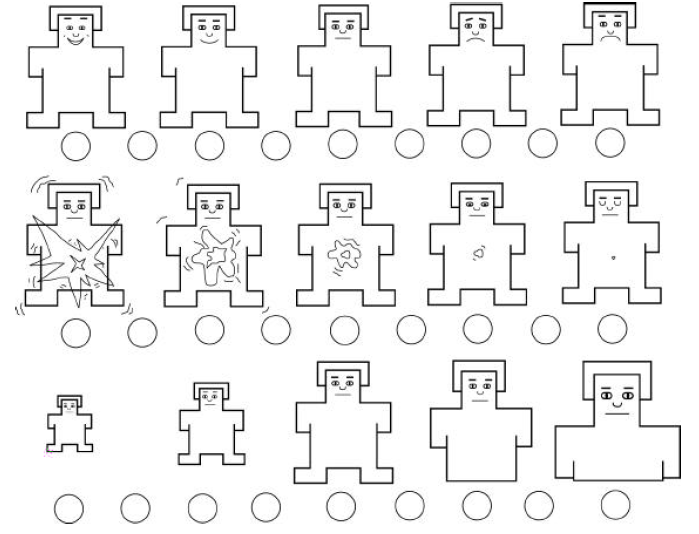


## Usability, Immersion and Engagement

You will see some statements about experiences. Please indicate, whether or not each statement applies to your experience. There are no right or wrong answers, only your opinions count. And please remember: Answer all these questions only referring to this one experience. Read through them to make sure you understand the statement. If you have any questions, please ask your administrator.

1. How well do you believe your performance in the simulator reflects your current power mobility skills (joystick control)?

|________|________|________|________|________|

| Not at all |  | Moderate |  | Extremely  Well |
| --- | --- | --- | --- | --- |

1. Did the tasks seem too easy or too difficult for your abilities?

|________|________|________|________|________|

| Too easy |  | Moderate |  | Too difficult |
| --- | --- | --- | --- | --- |

1. Do you feel that your power mobility skills improved during the simulator session?

|________|________|________|________|________|

| No  improvement |  | Moderate |  | Significant improvement |
| --- | --- | --- | --- | --- |

1. After using the simulator, do you feel more confident in handling a power wheelchair?

|________|________|________|________|________|

| Not at all |  | Moderate |  | Very Confident |
| --- | --- | --- | --- | --- |

1. How aware were you of the real world surrounding while navigating in the virtual world? (i.e. sounds, room temperature, other people, etc.)?

|________|________|________|________|________|

| Extreme  aware |  | Moderate |  | Not aware at all |
| --- | --- | --- | --- | --- |

1. I had a sense of acting in the virtual space, rather than operating something from outside.

|________|________|________|________|________|

| Strongly Disagree | Disagree | Neutral | Agree | Strongly Agree |
| --- | --- | --- | --- | --- |

1. How much did your experience in the virtual environment seem consistent with your real-world experience?

|________|________|________|________|________|

| Not consistent at all |  | Moderate |  | Extremely consistent |
| --- | --- | --- | --- | --- |

1. In the computer generated world, I had a sense of "being there".

|________|________|________|________|________|

| Not at all |  | Moderate |  | Very much |
| --- | --- | --- | --- | --- |

1. I felt present in the virtual space.

|________|________|________|________|________|

| Strongly Disagree | Disagree | Neutral | Agree | Strongly Agree |
| --- | --- | --- | --- | --- |

1. I would imagine that people would learn to use the wheelchair training simulator very quickly.

|________|________|________|________|________|

| Strongly Disagree | Disagree | Neutral | Agree | Strongly Agree |
| --- | --- | --- | --- | --- |

1. I found the system very difficult to use.

|________|________|________|________|________|

| Strongly Disagree | Disagree | Neutral | Agree | Strongly Agree |
| --- | --- | --- | --- | --- |

1. I needed to learn a lot of things before I could get going with the system.

|________|________|________|________|________|

| Strongly Disagree | Disagree | Neutral | Agree | Strongly Agree |
| --- | --- | --- | --- | --- |

1. Was the simulator easy to understand and navigate?

|________|________|________|________|________|

| Too easy |  | Moderate |  | Too difficult |
| --- | --- | --- | --- | --- |

1. Did the interface feel user-friendly and intuitive?

|________|________|________|________|________|

| Not at all |  | Moderate |  | Extremely  Well |
| --- | --- | --- | --- | --- |

1. Were the tasks and instructions clear and easy to follow?

|________|________|________|________|________|

| Too easy |  | Moderate |  | Too difficult |
| --- | --- | --- | --- | --- |

1. How satisfied are you with your experience using the simulator?

|________|________|________|________|________|

| Not at all |  | Moderate |  | Extremely  Well |
| --- | --- | --- | --- | --- |

1. Would you recommend the simulator as a training tool for new power wheelchair users?

|________|________|________|________|________|

| Not at all |  | Neutral |  | Extremely  Recommend |
| --- | --- | --- | --- | --- |

1. Would you recommend the simulator as assessment tool for new wheelchair users?

|________|________|________|________|________|

| Not at all |  | Neutral |  | Extremely  Recommend |
| --- | --- | --- | --- | --- |

## Semi-structure Interview Section (exploring usability and potential applications of the wheelchair simulator system)

**Section Introduction:**

Thank you for participating in our research on the Wheelchair Simulator project. Our study aims to explore and validate how virtual wheelchair simulator can be used as effective tool for assessing and training both power mobility skills and cognitive abilities. By sharing your feedback, you are contributing to refining this technology to better meet the needs of wheelchair users and healthcare professionals in real-world applications.

**For Wheelchair users:**

We’re interested in hearing your thoughts on the simulator, including your suggestions, opinions, and experiences using it. Specifically, we’d like to understand how well it supports the practice and improvement of skills that are essential for everyday wheelchair use.

**For healthcare professionals:**

Your insights are valuable in helping us understand the simulator’s potential as a clinical tool for assessment, training, and integration into protocols for wheelchair prescription and training. Your feedback on its practical utility and any suggestions for refinement are highly appreciated.

The interview will be recoded solely for research purposes, so we capture all important information, and we assure you of complete confidentially and privacy. There are no right or wrong answers – just open and honest insights.

Questions and Follow-up Prompts:

1. What aspects of the simulator did you find most helpful for assessing or practicing wheelchair skills?

- *Follow-up:*
  - *Can you describe a specific skill that was particularly well-supported by the simulator?*
  - *How do you feel the simulator helps with skills you find challenging in daily life?*

1. What changes or improvements would you suggest for the simulator to better meet user needs?

- *Follow-up:*
  - *Are there any specific features or controls you would like added or modified?*
  - *Did you notice any aspects that seemed overly complicated or not intuitive?*
  - *Follow-up: How could the simulator’s [visual or sensory] feedback be improved?*

1. Do you see value in using wheelchair simulators for training or assessment? If so, in which specific situations?

- *Follow-up:*
  - *Do you feel there are certain environments (e.g., home, urban or clinical settings) where simulation would be especially helpful?*
  - *In what ways could this simulator support or replace traditional training methods?*
  - *Could you see this as useful in a rehabilitation or clinical setting? Why or why not?*

1. Do you believe that new users can effectively learn skills through the simulator?

- *Follow-up:*
  - *Are there specific skills that might be easier or harder for new users to learn with the simulator?*
  - *How confident are you that the simulator could help new users transition to real-world wheelchair use?*
  - *What adjustments could make the simulator more supportive for first-time users?*

1. Could you rank the following Simulator’s system metrics in terms of their importance for evaluating the effectiveness of wheelchair simulator-based training and assessment?

| Metric | Definition | Importance Ranking (1-5) | Relevance Rating  (1-5) | Note |
| --- | --- | --- | --- | --- |
| Task Completion Time | The time taken to complete a specific task or sequence within the simulation. |  |  |  |
| Error Rate (Collisions) | The number of times the participant collides with obstacles, walls, or other elements in the simulated environment |  |  |  |
| Path Efficiency (deviation from the optimal path) | Measures the difference between the path taken by the participant and the pre-defined optimal or shortest path. |  |  |  |
| Manoeuvrability (number of commands) | The total number of control inputs (e.g., forward, reverse, turn commands) made during a task. |  |  |  |
| Hand Control (Smoothness Levels) | Measures the fluidity and steadiness of the participant’s control inputs (e.g., joystick or other control device) during navigation. |  |  |  |
| Hear Rate Variability (Stress Indicators) | Variability in heart rate measured throughout the simulation, often calculated using the root mean square of successive heartbeat differences (RMSSD) or the standard deviation of inter-beat intervals (SDNN). Higher variability can indicate relaxation, while lower variability can suggest stress. |  |  |  |
| Skin Conductance (Arousal levels) | Measures the electrical conductance of the skin, which changes with sweat gland activity associated with arousal. This is measured continuously and averaged across tasks to provide a measure of arousal, with higher values indicating higher arousal levels. |  |  |  |
| Eye Tracking (Fixation and saccades) | Measures the participant's gaze behaviour, recording the number and duration of fixations (moments where gaze remains on a specific point) and saccades (quick movements between points). This is computed through eye-tracking technology, providing data on the participant’s attention and focus on task-relevant areas. |  |  |  |
| Head Movements (Times of not at looking at the simulator environment) | Counts instances when the participant’s head turns away from the simulator’s environment. Computed by tracking head orientation; each time the participant’s gaze leaves the screen is recorded. Higher counts may indicate disengagement or distraction. |  |  |  |

We have finished with the questions; is there anything else you would like to add, or are there any additional suggestions you have for the system?

Thank you for your time. Please don’t hesitate to reach out if you have any further comments or thoughts in the future.

## Assessments Overview

| Section Name | Themes/Assessments | Questions/Tasks | Description |
| --- | --- | --- | --- |
| Pre-Experience | Demographics Information | 1.1 | Gathers background information like age, gender, wheelchair use duration, type, joystick type, dominant hand and diagnosis |
|  | Wheelchair Skills Test Questionnaire (WST-Q) | 1.2 | Standardized assessment of wheelchair skills (download link provided) |
|  | Montreal Cognitive Assessment (MOCA) Version 8.1 English | 1.3 | Standardized assessment of cognitive function (download link provided) |
| During Experience | PAAS Scale | 2.1 | Measures perceived mental effort and difficulty during tasks (scale provided) |
|  | Power Mobility Road Test Assessment Sheet | 2.2 | Scores performance on specific wheelchair manoeuvring tasks (scoring criteria provided) |
| Post-Experience | NASA-TLX (Overall Cognitive Workload) | 3.1 | Measures subjective workload across six factors (scale provided) |
| Post-Experience | Self-Assessment Manikin (SAM) | 3.2 | Rates emotions on three scales: valence (pleasantness), arousal (excitement), and dominance (control) |
|  | Usability, Immersion and Engagement | 3.3 | Likert-scale questions on user experience aspects |
|  | Semi-structured Interview | 3.4 | Explores user thoughts and suggestions on the simulator's usability and potential applications |
